# A Structural Framework for a Near-Minimal Form of Life: Mass and Compositional Analysis of the Helical Mollicute *Spiroplasma melliferum* BC3

**DOI:** 10.1371/journal.pone.0087921

**Published:** 2014-02-21

**Authors:** Shlomo Trachtenberg, Peter Schuck, Terry M. Phillips, S. Brian Andrews, Richard D. Leapman

**Affiliations:** 1 Dept of Microbiology and Molecular Genetics, Institute for Medical Research Israel-Canada, The Hebrew University-Hadassah Medical School, Jerusalem, Israel; 2 Laboratory of Neurobiology, National Institute of Neurological Disorders and Stroke, National Institutes of Health, Bethesda, Maryland, United States of America; 3 Laboratory of Cellular Imaging and Macromolecular Biophysics, National Institute of Biomedical Imaging and Bioengineering, National Institutes of Health**,** Bethesda, Maryland, United States of America; Miami University, United States of America

## Abstract

*Spiroplasma melliferum* is a wall-less bacterium with dynamic helical geometry. This organism is geometrically well defined and internally well ordered, and has an exceedingly small genome. Individual cells are chemotactic, polar, and swim actively. Their dynamic helicity can be traced at the molecular level to a highly ordered linear motor (composed essentially of the proteins fib and MreB) that is positioned on a defined helical line along the internal face of the cell’s membrane. Using an array of complementary, informationally overlapping approaches, we have taken advantage of this uniquely simple, near-minimal life-form and its helical geometry to analyze the copy numbers of *Spiroplasma*’s essential parts, as well as to elucidate how these components are spatially organized to subserve the whole living cell. Scanning transmission electron microscopy (STEM) was used to measure the mass-per-length and mass-per-area of whole cells, membrane fractions, intact cytoskeletons and cytoskeletal components. These local data were fit into whole-cell geometric parameters determined by a variety of light microscopy modalities. Hydrodynamic data obtained by analytical ultracentrifugation allowed computation of the hydration state of whole living cells, for which the relative amounts of protein, lipid, carbohydrate, DNA, and RNA were also estimated analytically. Finally, ribosome and RNA content, genome size and gene expression were also estimated (using stereology, spectroscopy and 2D-gel analysis, respectively). Taken together, the results provide a general framework for a minimal inventory and arrangement of the major cellular components needed to support life.

## Introduction

Spatial and temporal order are reflections of the development, structure and physiology of living systems. A promising and fundamental approach to understanding cell function aims to develop a continuous map of this order, from single molecules to living cells, taking advantage of a carefully chosen simple, yet fully functional and spatially well-defined, model cell. Bacteria recommend themselves for this purpose, as they are the simplest of unicellular organisms, with a hierarchy of complexity largely reflected by genome size. The genome specifies the blueprint of the cell through temporal and environmental control of gene expression, which in turn specifies its protein inventory. However, the cell itself is, at any moment, more complex than the sum of its gene products due to post-translational modifications and three-dimensional compartmentalization.

A cellular system may evolve progressively from a simpler state; alternatively, a complex system may be reduced to a simpler one by regressive evolution. The mollicutes – mycoplasmas, spiroplasmas and acholeplasmas – are examples of the latter. The mollicutes’ genome reduction followed the transition to parasitism during which various physiological and structural systems became redundant [1], [2]. In the helical spiroplasmas, the average genome is only ∼1.30×10^6^ bp [3], [4]. Spiroplasmas [5], [6] are well defined geometrically and well ordered internally [7], [8]. They are chemotactic [9]–[11], polar, and active swimmers [12], [13]. Their dynamic helicity [12], [14] can be traced at molecular dimensions [15], [16] to a highly ordered linear motor – a flat, monolayered ribbon assembled from parallel fibrils – that is attached to the inner surface of the tubular cell’s only membrane along the shortest helical line [17]–[21]. Tube and motor are mutually coiled into a dynamic helix [14]. This helical symmetry renders the cell amenable to detailed quantitative geometrical analysis because the properties of short cellular segments or extracted structural components can be extrapolated to the whole cell. Furthermore, the cell’s helical symmetry allows for merging of complementary light microscopy, electron microscopy, and electron crystallography data at the cellular and molecular levels. The resulting structural framework can be further filled with complementary data obtained from other analytical techniques. The small genome [22]–[25], relative structural simplicity and amenability to both experimental manipulation and theoretical/computational analyses interface the mollicutes with the forefront of synthetic biology [26]–[28].

Here we extend earlier studies on the structure of *Spiroplasma*, its dynamic cell geometry and its molecular origins [14], [15], [19], to the spatial and compositional organization of the whole cell and its parts. Using scanning transmission electron microscopy (STEM) [16], [29], [30], we determine (*i*) the mass-per-length of whole intact cells, (*ii*) the mass-per-length of isolated cytoskeletons and cytoskeletal fibrils, and (*iii*) the mass-per-area of cytosol-free cells (*i.e.,* membrane envelopes). We then fit (*iv*) the mass density data into the geometrical framework constrained by the helical symmetry parameters of an average live cell as obtained by dark field, phase contrast and video light microscopy [12]. Next, we measure (*v*) the sedimentation and diffusion coefficients of live cells so that hydrodynamic, geometrical and mass data can be combined to determine Spiroplasma’s water content. We also measure (*vi*) the bulk chemical composition of intact cells in terms of relative fractions of proteins, lipids, carbohydrates, DNA and RNA, and subsequently normalize these data to an average cell. Concerning the cytosol, we estimate (*vii*) the number density of ribosomes–the only internal structural features visible in the cell– by using stereology and tomography. We further estimate (*viii*) the genome size from measured DNA content per cell, and assess (*ix*) cellular RNA content based on cytosolic ribosome density and direct spectroscopic measurements. Lastly, we estimate (*x*) gene expression of *Spiroplasma* by 2D-gels of the different cell fractions.

## Results

### 1. Geometrical Parameters of a *Spiroplasma* Cell

**Figure 1:**
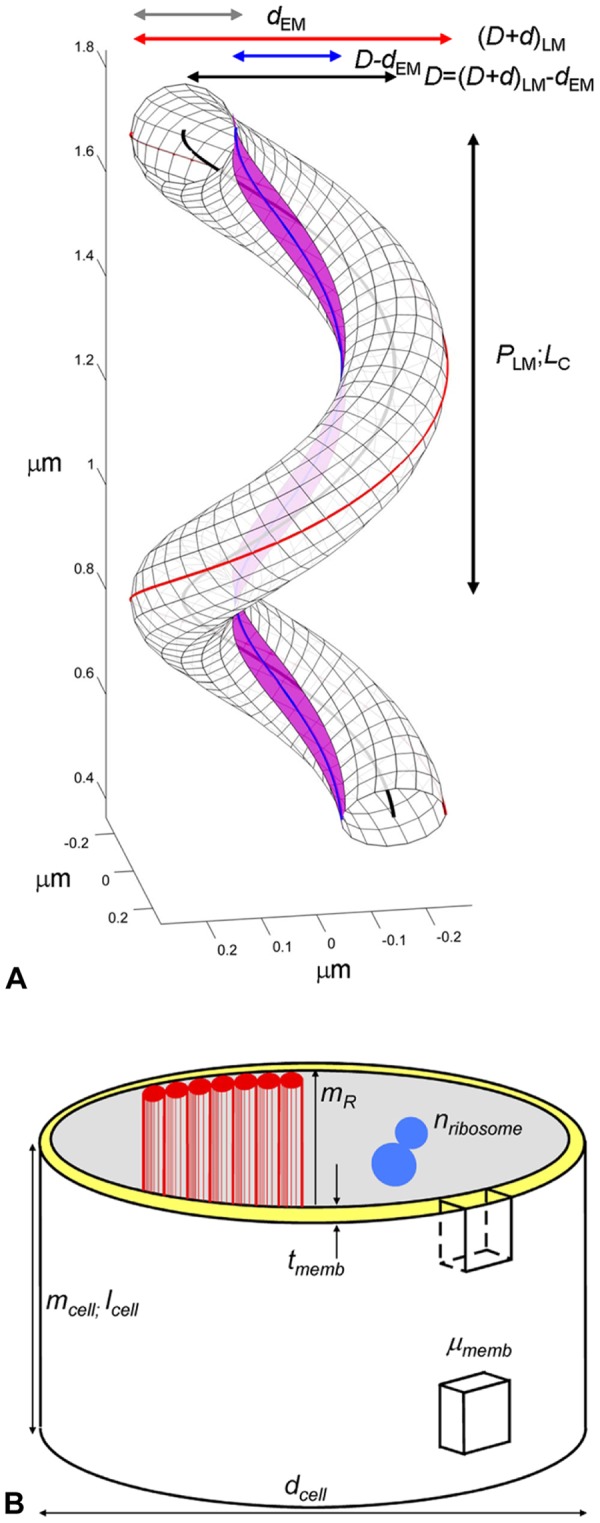
Cell geometry and measurble parameters. **[A]** Schematic diagram showing a right-handed, 1.5 turn helical tube with dimensions similar to an average *Spiroplasma* cell. LM and EM subscripts indicate parameters measured directly by light- and electron microscopy, respectively. one helical repeat (*P*; vertical black arrow) and corresponding helical centerline, *L_C_–*black line, are marked. The basic tube diameter (*d;* grey horizontal arrow), tube centerline diameter (*D;*black horizontal arrow), shortest line, *L_S_*–blue line–and corresponding inner tube diameter (*D-d;*blue arrow) and outer coiled-tube diameter (*D+d;*red arrow) are labeled. The center of the cytoskeletal ribbon, *L_R_*, (magenta) follows the geometrically shortest helical line (blue). See **Eqs. 2, 17** and **18** for analytical details [14] and **Table 1** for actual dimensions. **[B]** *Spiroplasma* cell compartments and measured STEM parameters are illustrated. The major mass compartment is a membrane tube of diameter d_cell_ and thickness *t_memb_* (yellow) (see **Fig. 7** and **Table 1**) to which a seven-fibered cytoskeletal ribbon (red) is attached. Together these form the dynamic cell envelope. The following parameters were determined on freeze-dried preparations: (i) the mass-per-length of whole, intact cells (*m_cell_*); (ii) the mass-per-area of patches of defined areas of empty membrane vesicles (*µ_memb_*); and (iii) the mass-per-length of isolated cytoskeletal ribbons (*m_R_*) and their component single fibrils (*m_fibril_*) [16]. Ribosome counts, *n_ribosome_*, per unit area of thin sections were used to estimate the number of ribosomes per cell (**Eqs. 22, 24**).

*Spiroplasma* is essentially helically symmetric, even though this symmetry is a transient state in the cell’s dynamics because in order to swim directionally in a low Reynolds number environment cells must continuously deviate from strict helical symmetry by a combination of, bending, hand-switching, and changing pitch [12]. Nonetheless, the tubular organization of *Spiroplasma* [14], [18] (i.e., its uniform mass per unit cell length, mass per unit membrane area, and mass per length of the cytoskeleton) can be used together with bulk chemical analyses to provide a model of an average helical cell. **Fig. 1A** is a geometrical model of a 1.5-turn segment of a right-handed, helical, tubular cell of diameter *d* (grey line); indicated parameters include the cell’s helical pitch (*P*), centerline (*L_C_*; black), diameter (*D*; black), outer coil diameter (*D+d*; red), and cytoskeletal ribbon, *L_R_*, (magenta) the center of which is situated here along the shortest, *L_S_*, helical line (blue). These parameters can be determined, directly or computationally, from a combination of light (LM) and electron (EM) microscopy images, which enables calculation of *Spiroplasma*’s three-dimensional helical geometry (for a full account see [14] ) and then integration of the geometrical model with the mass data presented in the following sections to obtain a biomolecular inventory of the cell. The helical parameters for an average cell, as well as the resulting length-per-area and mass-per-area results, normalized to an average helical repeat and average cell, are summarized in **Table 1**.

**Table 1:** Geometric Parameters of *Spiroplasma* Cells.

Parameters	Mean	SD	Units	Reference
**Geometric parameters**				
Coil outer diameter (*D*+*d*)	570	70	nm	Fig. 1
Tube diameter (*d*)	188	19	nm	Fig. 1
Coil centerline diameter (*D*)	382	69	nm	Fig. 1
Coil inner diameter (*D-d*)	194	29	nm	Fig. 1
Helical pitch (*P*)	889	118	nm	Fig. 1
Number of turns (*n*)	4	0.4		Fig. 1
Centerline lengthper turn (*L_C_*)	1,490	210	nm	Eq. 2, Fig.1
Centerline lengthper cell (*L_cell_*)	5,950	1033	nm	Eq. 3
Cell axial length (*nP*)	3,510	593	nm	Fig. 1
Axial ratio (r)	6.24	1.46		Eq. 1
Helical repeat volume (*V_C_)*	4.10×10^7^	6.00×10^6^	nm^3^	Eq. 4
Cell volume (*V_cell_*)	1.65×10^8^	0.23×10^8^	nm^3^	Eq. 5
Helical repeat surface (*S_C_)*	8.90×10^5^	1.24×10^5^	nm^2^	Eq. 15
Cell surface area (*S_cell_*)	3.54×10^6^	0.61×10^6^	nm^2^	Eq. 16
**Mass parameters**				
Cell mass-per-length (*m_cell_*)	3.74	0.68	MDa/nm	Eq. 6, Figs. 1,3
Mass per cell (*M_cell_*)	22.2	5. 4	GDa	Eq. 7
Membrane mass-per-area(*µ_memb_*)	4.67	0.70	kDa/nm^2^	Figs. 1, 6
Membrane mass per cell(*M_memb_*)	1.66	0.38	GDa	Eq. 17
**Ribosome parameters**				
Number per unit area (*n_ribosome_*)	962	102	µm^−2^	Eq. 24, Fig. 1
Number per unit volume(*ν_ribosome_*)	8,754	935	µm^−3^	Eq. 24
Number per cell (*N_ribosome_*)	1,440	159		Eq. 24
Unit mass of ribosome(*m_ribosome_*)	2.7	0.05	MDa	
Mass per cell (*M_ribosome_*)	3.89	1.06	GDa	Eq. 27
**Cytoskeleton mass parameters**				
Length of ribbon per turn(*l_R_* = *l_S_*)	1,092	179	nm	Eq. 19, Fig. 1
Length of ribbonper cell (*L_R_*)	4,368	716	nm	Eq. 20
mass-per-length (*m_R_*)	189	70	kDa/nm	
Mass per cell (*M_R_*)	8.26×10^5^	3.49×10^5^	kDa/nm	Eq. 21

Geometric parameters of *Spiroplasma* cells were measured directly from high-intensity, dark-field light microscopy and cryoelectron microscopy. Using the helical symmetry of the cell, parameters were extrapolated to entire cells. STEM mass data, obtained per unit length or area, were similarly extrapolated to whole cells. **Fig. 1** illustrates diagramatically several of these parameters.

The following standard nomenclature is used throughout: (*i*) lower case refer to mass-per-unit-length (Da/nm) with descriptive subscripts, e.g., *m_cell_* = mass-per-length of cell; (*ii*) Greek letters refer to mass-per-unit-area (Da/nm^2^) with descriptive subscripts, e.g., *µ_memb_* = mass-per-area of membrane; and (*iii*) upper-case letters refer to quantity-per-cell with descriptive subscripts, e.g., *M_cell_* = mass of cell. Quantities referring to one helical repeat are marked with a subscript C, e.g., *L_C_* = length per helical repeat.

The axial ratio, an important hydrodynamic parameter (see **Results section** **3**), of *Spiroplasma*’s helical shape is given by
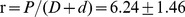
(1)(All errors, throughout, are stated as SD, Standard Deviation; for statistic analysis see **Materials and Methods** **section 8**)

The length of the centerline *L_C_* per helical repeat *P* is (see **Fig. 1A**)
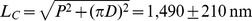
(2)and for a cell consisting of *n* = 4 helical turns, the centerline or contour length *L*_cell_, is
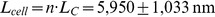
(3)the volume per helical repeat is
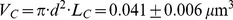
(4)and for a cell with n = 4 helical turns
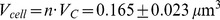
(5)


### 2. Dry mass of intact cells

Since *Spiroplasma* cells are coiled tubes with helically geometry [14], their entire structure can in principle be built by invoking lengthwise uniformity [18] and extending a fixed segment along the helical centerline, as indicated in **Fig. 1A**. A simplified schematic representation of a cellular segment illustrating the compartments that are measurable by STEM is presented in **Fig. 1B**. The major component of the mass consists of the membrane tube (shown in yellow) to which the cytoskeletal ribbon is attached (red). Cytoplasm, chromosomal DNA, and ribosomes with associated RNA are the major components that fill this tube. When cells are lysed through a combination of osmotic shock and ultrasonication, the contents are released leaving behind empty vesicles. Measured mass parameters for these membrane preparations are defined and presented in **Table 1**. Subsequently, we take advantage of the helical symmetry to extrapolate these complementary data to determine the total mass of a representative average cell (**Table 1** **)**.

**Figure 2:**
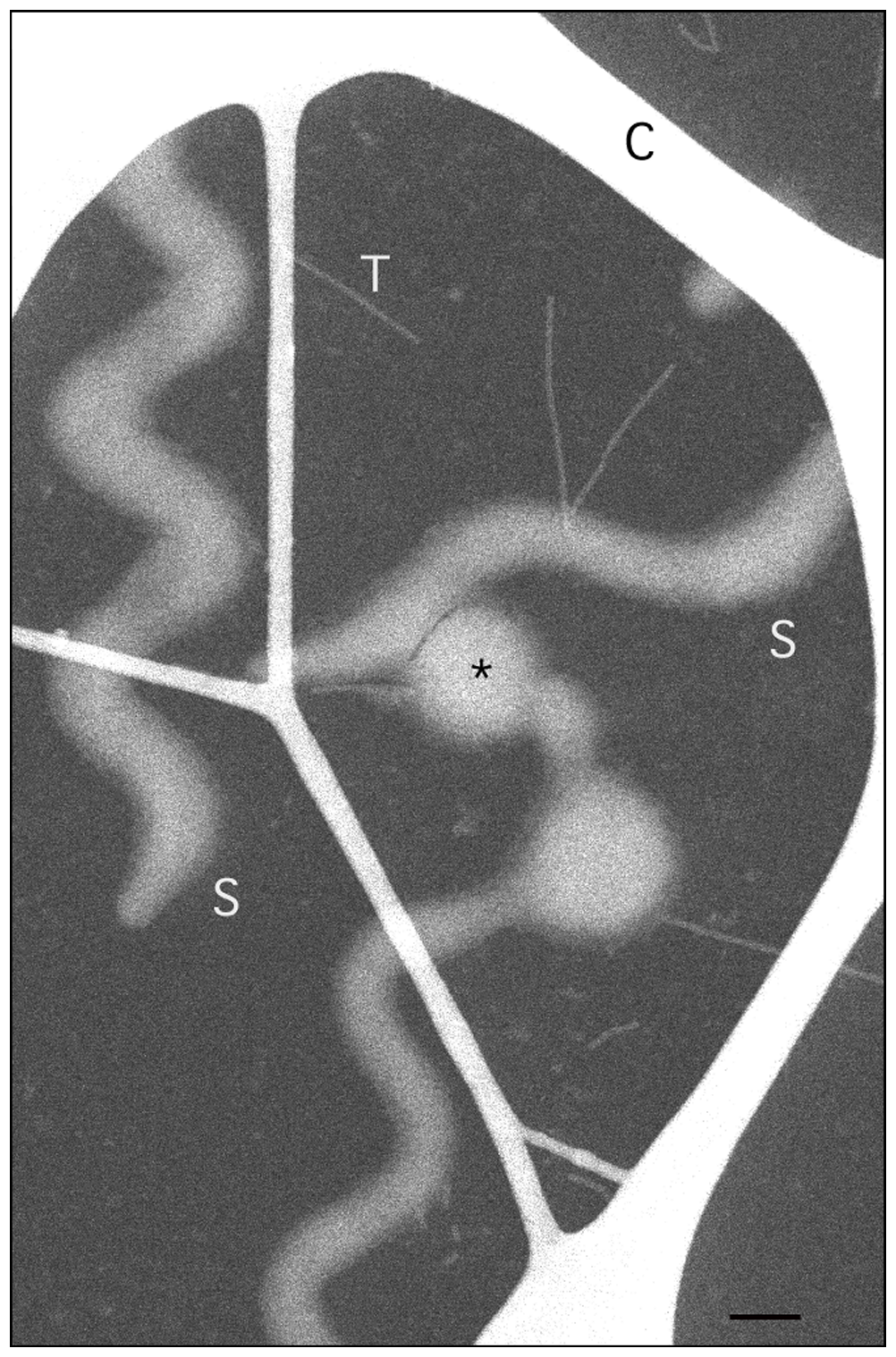
STEM dark field image of freeze-dried, intact *Spiroplasma* cells. A thick carbon lace (C) supports a thin (∼3 nm) carbon film. Cells (S) and TMV particles (T), the latter used for mass calibration, are scattered on the carbon support. The cells are polar and have distinct tapered (left edge) or round ends (not seen). Older cells are known to vesiculate, as indicated by the asterisk. Note the difference in mass, as reflected by differences in image brightness, between the straight, uniform tubular cell segments (dimmer) and the heavier (brighter) inflection points of the collapsed coils. Only the straight, uniform segments were used for mass measurements. Scale bar = 200 nm.

**Figure 3:**
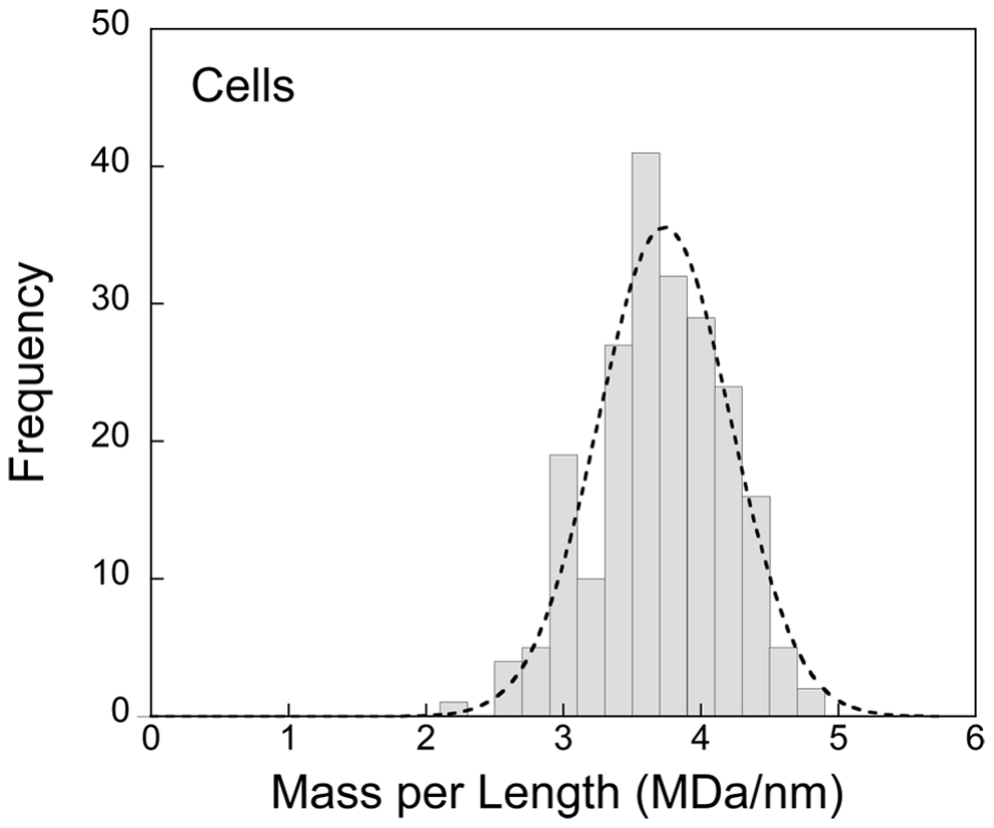
Histogram of the mass-per-length distribution in MDa/nm for a *Spiroplasma* cell population. The Gaussian (r^2^ = 0.97) fit superimposed on the histogram implies a normal distribution. The average mass is 3.74±0.68 (SD) MDa/nm. The mass of an average cell is 22.2 GDa.

Dark-field STEM images of *Spiroplasma* cells (**Fig. 2**) demonstrate the level of spatial preservation achieved in our preparative procedures; it is evident that cellular margins are regular and well delineated. The projected width of the cell is uniform along its length and similar to image data from vitrified cells. Following the method introduced by Wall et al. [29], addition of tobacco mosaic virus (TMV) particles to the specimen of *Spiroplasma* cells provides an ideal standard for STEM mass determination. The structure of TMV has been determined to atomic resolution, and their uniform, compact structure, consisting of 18-nm diameter rods of length 300 nm and total mass 39.3 MDa, is dimensionally stable under EM conditions. We find that 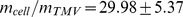, where 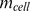 is the mass-per-length of the *Spiroplasma* cell tube, and 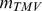 is the mass-per-length of TMV. Taking the accepted value of/ = 0.131 MDa/nm, we calculate the mass per unit length of a straight tubular *Spiroplasma* segment (**Fig. 3**) as:
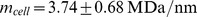
(6)


The uncertainty of average cell mass-per-length is much smaller than indicated by the standard deviation (SD) of the individual measurements from tube segments indicated above and in **Materials and Methods** **Section 8**. and also evident in the histogram presented in **Fig. 3**.

From the mean contour length of the cell *L_cell_*, we can estimate the average cell mass as
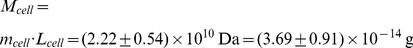
(7)where we divide the mass in Daltons by Avogadro’s number/mass units per gram to convert the cell mass to grams.

We next calculate the dry mass fraction of a *Spiroplasma* cell from the dried cell mass-per-length and the cell volume-per-length (*i.e*., cell cross-sectional area); the dry mass fraction can then be used to estimate the cell’s hydration and density. The density of the dry mass component is given by:
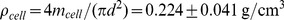
(8)


### 3. Cell Hydration

The STEM mass measurements presented above are made on freeze-dried specimens, and therefore do not provide direct information about the state of hydration of *Spiroplasma*. STEM mass measurements of vitrified hydrated specimens are complicated by the embedding ice layer, which must be uniform and of the same thickness as the cells in order to obtain useful data [31]; such conditions are impractical for objects as large as *Spiroplasma*.

To obtain independent information about the hydration state of *Spiroplasma*, which complements structural and mass data obtained by microscopy, we carried out dynamic light scattering (DLS) and analytical ultracentrifugation (AUC) experiments on live cells. Although these biophysical techniques are not commonly employed to study such large objects as live bacteria, we did not encounter difficulties in performing sedimentation experiments provided that very low rotor speeds were used. The interference optics and the analysis techniques based on the ls-g*(s) method are discussed in Materials and Methods. Measurements were made on *Spiroplasma* cells that were starved and maintained at suboptimal temperature, and therefore not actively swimming or replicating. Under these conditions, both DLS scattering and AUC are governed by the same translational coefficient of friction, as required by the Svedberg equation in the case of AUC.

**Figure 4:**
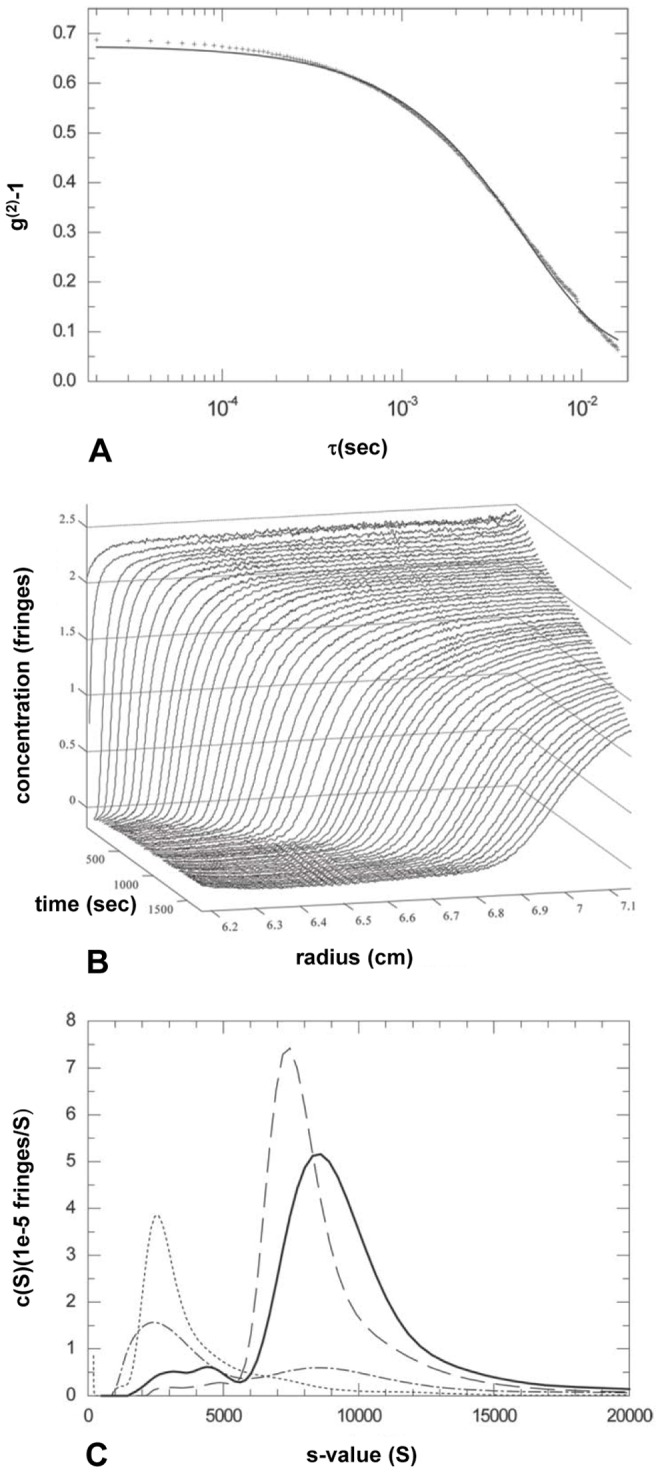
Hydrodynamic studies of a *Spiroplasma* cell population. **[A]** Dynamic light scattering of live *Spiroplasma* cells in isotonic PBS. Shown is the autocorrelation g^(2)^-1 function acquired at 90° (crosses) overlaid with the best-fit single species fit (solid line). The data estimate an average diffusion constant of 6.1×10^−9^ cm^2^/sec. **[B]** Evolution of concentration profiles across the Spiroplasma sample in PBS at various times after the start of centrifugation at 3,000 rpm. **[C]** Sedimentation coefficient distributions calculated by least-squares modeling of the concentration distributions in **[B]** by superposition of Lamm equation solutions of non-diffusing species, ls-g*(s). Shown are the distributions obtained at *Spiroplasma* concentrations of 0.015 mg/ml (dash-dotted line), 0.15 mg/ml (solid line), 1.5 mg/ml (dashed line) and buffer (dotted line).

Dynamic light scattering revealed a relatively broad size-distribution, with a peak diameter of 400 nm and an average diameter of ∼700 nm, corresponding to a translational diffusion coefficient of 6.1×10^−9^ cm^2^/s (**Fig. 4A**). Thus, on the time-scale of the sedimentation experiment (**Fig. 4B**), the extent of diffusion is negligible and the sedimentation coefficient distribution, as determined by the ls-g*(s) technique, directly reflects the polydispersity of the cells in suspension (**Fig. 4C**). A major peak, representing intact single cells, is observed at a viscosity-corrected *s*-value of 9,930 S. There are also significant populations of broadly distributed larger species visible in the long tail toward higher s-values (≤20,000 S; likely aggregates) as well as some smaller species (in the range 3,000 to 4,000 S; probably fragments or dividing cells).

From these values, we can calculate the total hydrated mass of the cell. The Svedberg equation gives an estimated buoyant mass of a single cell as:
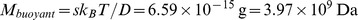
(9)where *k_B_* is Boltzmann’s constant, s is the sedimentation coefficient (in Svedberg units = 10^−13^ s), *T* is the temperature in Kelvin (293 K), and *D* is the diffusion coefficient (6.1×10^−9^ cm^2^/s). The estimated error in the determination of the sedimentation coefficient was less than 5%, which gives the buoyant mass of an average *Spiroplasma* cell as 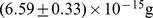, with an estimated error of less than 5%.

In calculating of the mass of the hydrated cell from the buoyant mass, the known volume can replace the need for a partial-specific volume usually required in the sedimentation analysis of macromolecules:
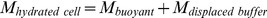
(10)where the mass of the displaced buffer can be determined from the product of the cell volume, 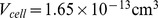, and the buffer density, 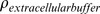, which has a known value of 1.00741 g/cm^3^. This gives a value for the average hydrated cell mass as:



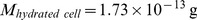
(11)By taking the ratio of the dry cell mass 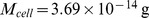 (obtained from STEM), and the hydrated cell mass (obtained from the AUC and DLS), we can estimate the dry mass fraction as:
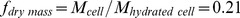
(12)which is comparable with the density of the dry mass component of the cell obtained from **Eq. 8**.

The solution experiments provide an estimate of the average hydrated cell density as:
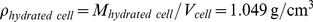
(13)


Subdividing the hydrated cell into contributions from water and dry constituents, we can calculate an average density of the dry mass as:
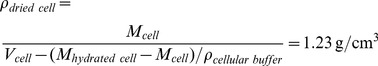
(14)where the density of the cellular buffer is assumed to be the same as the extracellular buffer used in the AUC experiments,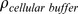 = 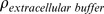.

It should be noted that the distinction between these two average densities from AUC data is unusual. Commonly only *ρ_dried cell_* is encountered in studies of proteins and other macromolecules. The ability to determine the average density including the water content is due to independent knowledge of the cell volume, and the large difference in values arises from the fact that the volume fraction occupied by water is quite significant. By considering typical densities for proteins, nucleic acids, carbohydrates, and lipids (approximately 1.37, 1.85, 1.65, and 1.10 gram/cm^3^, respectively) [32], we see that the dry component’s low density of 1.23 g/cm^3^ is consistent with the observation in the next section that the lipid-rich membrane accounts for a large fraction of *Spiroplasma* dry mass (**Section 4,** **Table 2**).

**Table 2:** Biochemical Analysis of Whole *Spiroplasma* Cells.

Analyte	Weight(µg)	SD(µg)	Percent	MethodsReference
**Total protein** 1	728.94	2.11	**71.3**	11.c. *Protein*
Unconjugatedprotein^2^	226.91	5.88	22.2	footnote 2
Lipoprotein	285.35	6.31	27.9	11.d. *Protein*
Glycoprotein	216.50	3.99	21.2	11.d. *Protein*
**Total lipid** 1	80.46	0.65	**7.9**	11.c. *Lipid*
Free lipid^3^	29.72	1.09	2.9	footnote 3
Fatty acid	16.92	0.82	1.7	11.d. *Lipid*
Phospholipid	3.66	0.37	0.4	11.d. *Lipid*
Cholesterol	22.84	1.01	2.2	11.d. *Lipid*
Glycolipids	7.31	0.26	0.7	11.d. *Lipid*
**Total carbohydrate** 1	82.61	0.49	**8.1**	11.c. *Carbohydrate*
Free carbohydrate^4^	12.98	0.99	1.3	11.d. *Carbohydrate*
**Total DNA**	41.06	0.06	**4.0**	11.c. *Nucleic acid* ***s***
**Total RNA**	88.21	0.85	**8.6**	11.c. *Nucleic acids*.

Total dry mass of analyzed cells was 1,022 µg. Individual values are given as absolute weights (in µg) within this total amount, as well as in percent of the total dry mass. *n* = 3 analyses per sample.

1 Total protein, lipid, and carbohydrate content are the sum of all subtypes.

2 Unconjugated protein is defined as pure protein or peptide free of detectable carbohydrate or lipid; it is derived by subtracting the sum of lipoprotein and glycoprotein from total protein.

3 Free lipid is defined as lipids with no detectable carbohydrate or protein; it is derived by subtracting the sum of lipid subtypes from total lipid.

4 Free carbohydrate is defined as carbohydrates with no detectable lipid or protein.

### 4. Mass of *Spiroplasma* Cell Membrane and Associated Components

*Spiroplasma* cells are enveloped by a single unit membrane [18], [19], as expected for bacteria of Gram positive origin. The *Spiroplasma* membrane is unusual in that it is relatively thick, contains cholesterol [33]–[35], and is densely covered externally with a unique 26 kDa lipoprotein named spiralin [33], [34]. External polysaccharides may also be present, as suggested by ruthenium red staining (data not shown). The cytoskeleton, which also serves as a motor known to contain the Fib protein [21], is firmly attached to the cytoplasmic face of the membrane [18], [19] through interfacing proteins, one of which is likely to be MreB [18], [20], [36].

**Figure 5:**
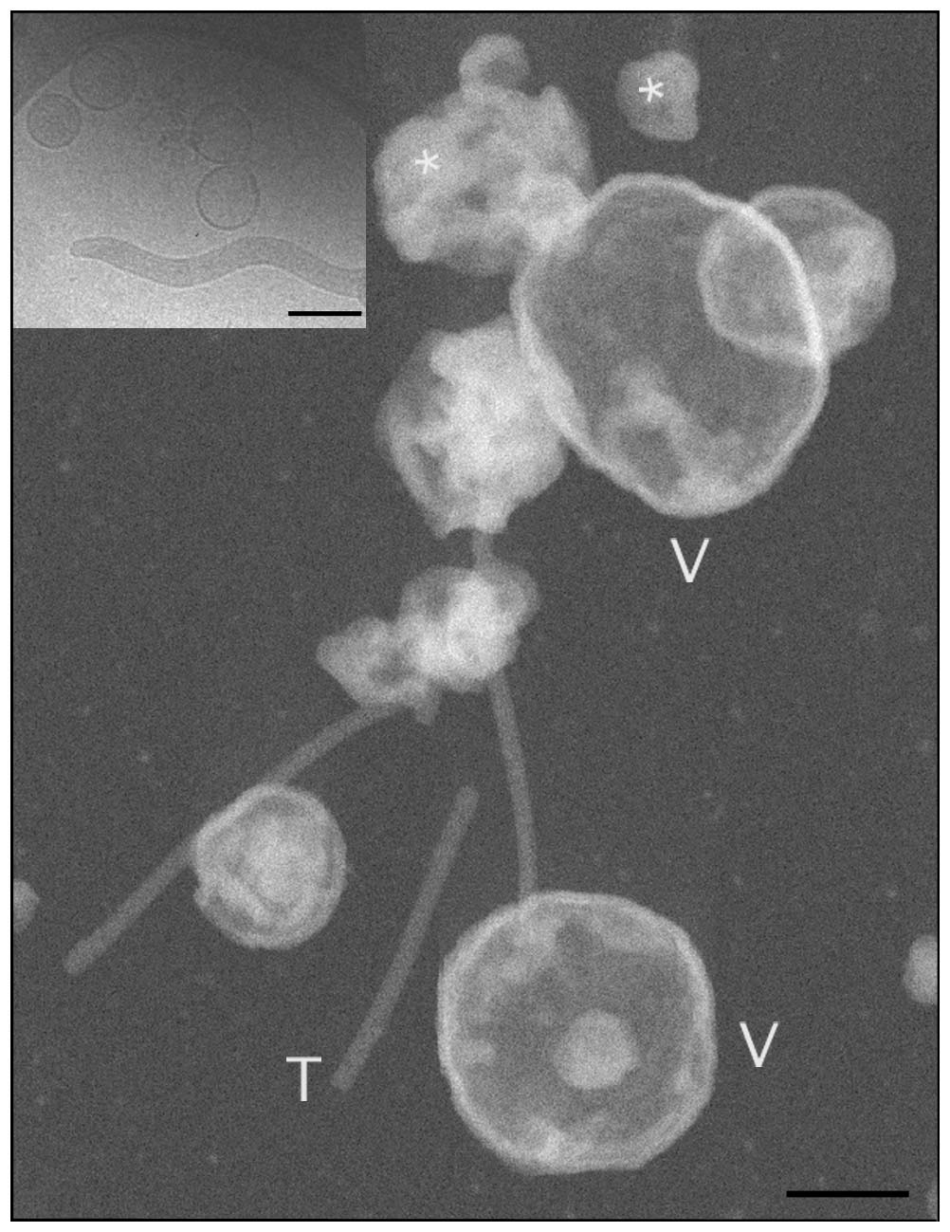
STEM, dark-field image of collapsed *Spiroplasma* vesicles on a thin carbon film. **[A]** Completely collapsed, flat vesicles are marked (V). Only uniform areas of these vesicles were used for mass-per-area measurements. Many small, tightly aggregated vesicles are scattered on the carbon film. TMV particles (T) were used as mass standards. Scale bar = 100 nm. **Inset:** Vitrified membrane vesicles and a vitrified cell, suspended over a hole in a carbon film, are shown for comparison with the freeze-dried specimen**.** Scale bar = 0.4 µm.

We have determined by direct STEM measurements the mass-per-area of *Spiroplasma* membranes using a preparation of isolated, collapsed membrane vesicles obtained by osmotic shock, ultrasonication, extensive washing to remove all cytoplasmic components, and ultracentrifugation (see Materials and Methods). RNA and DNA were undetectable in this vesicle preparation, suggesting reliable removal of non-membrane bound components. A dark-field STEM image in Fig. 5A shows rapidly frozen, freeze-dried vesicles (V) and TMV particles (T) suspended over a thin carbon film. Large vesicles are seen to collapse to form a double membrane layer in their center, whereas smaller vesicles aggregate into a condensed, irregular mass of wrinkled layers (asterisks). Mass analyses were only performed on uniform central areas of large vesicles. For comparison, Fig. 5B shows an image of similarly prepared frozen-hydrated vesicles, which have large diameters, smooth boundaries, and which appear empty.

**Figure 6:**
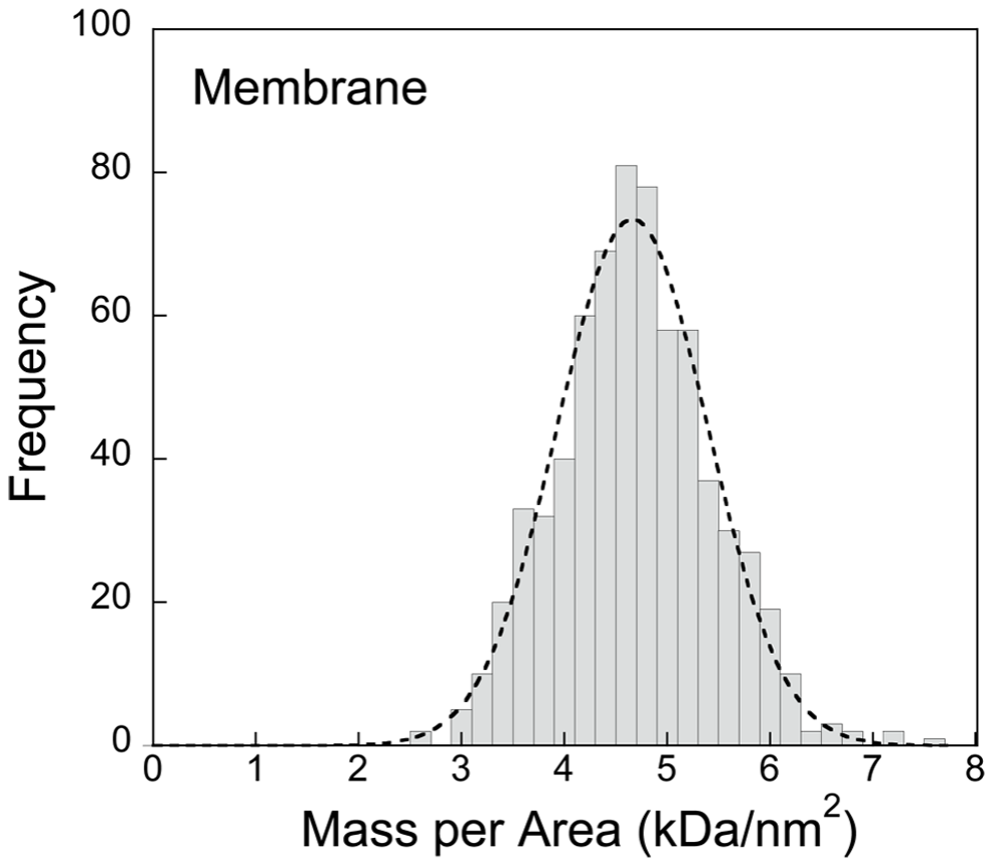
Histogram of the mass-per-area distribution for a collapsed, flat population of *Spiroplasma* membrane vesicles. A Gaussian fit (r^2^ = 0.97) is superimposed on the histogram suggesting a normal distribution. The average mass density per unit area of membrane is 4.67±0.70 kDa/nm^2^ (SD).

A histogram of the distribution of STEM mass-per-area measurements *µ_memb_* of *Spiroplasma* membranes is shown in **Fig. 6**. The data were normally distributed as indicated by the Gaussian fit with a mean of *µ_memb_*_ = _4.67±0.70 kDa/nm^2^.

The surface area of the membrane per helical repeat is given by
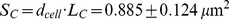
(15)


The surface area of the membrane per cell with n = 4 helical repeats is given by
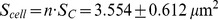
(16)


The total mass of the cell membrane, 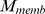 is therefore given by:
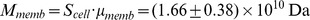
(17)


The mass fraction of *Spiroplasma* that is in the membrane and associated structures 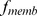 is given by:
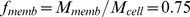
(18)


**Figure 7:**
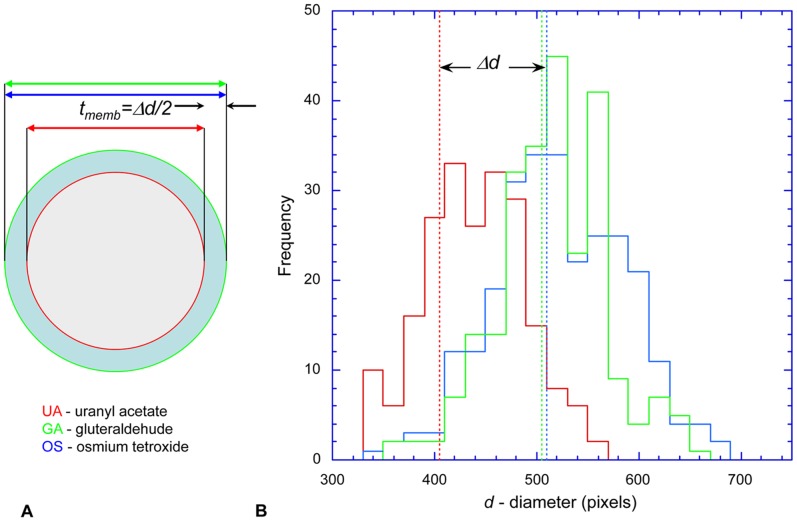
Estimating the cell membrane thickness. **[A]** The membrane of cells freeze-substituted in acetone/uranyl acetate (UA, red) is not contrasted, whereas the cytoskeleton is highly contrasted, so that its interface (red line) with the cytoplasm (gray) is sharply resolved. In cells freeze-substituted in acetone/osmium tetroxide (OS, blue) or acetone/glutaraldehyde (GA, green), the membranes themselves (light blue ring) are highly contrasted. The difference (*Δd*/2) in projected diameters of circular cross-sections (arrows) of differentially stained cells is a good estimate of membrane thickness, *t_memb_*. **[B]** Histograms of diameters of circular cross-sections of cells freeze-substituted in uranyl acetate (UA, red), osmium tetroxide (OS, blue) and glutaraldehyde (GA, green) are shown. The average diameters of the GA and OS specimens are very similar – 504±53 and 511±63 pixels (SD), respectively – whereas that of UA – 403±40 – is smaller. The difference (*Δd*) indicated by the double-headed arrow implies that the membrane thickness, *t_memb_*, is ∼ 0.2*d*/2. The pixel size is 0.39 nm. See **Fig. 1B.**

As previously noted, the *Spiroplasma* cell envelope is unusually thick, at least partly due to a dense coating of the lipoprotein spiralin in addition to polysaccharides. The membrane of mollicutes contains a larger proportion of integral proteins compared to walled bacteria [37]. The thickness of the cell envelope was estimated by a differential freeze-substitution approach [18], [19] (data not shown)**,** which is depicted schematically in **Fig. 7A**. In brief, the membrane and cytoplasm of *Spiroplasma* cells freeze-substituted in acetone/uranyl acetate (UA) are not contrasted, whereas the cytoskeletal elements, tightly adhering to the inner aspect of the membrane, are highly contrasted. Thus, the interface of the cytoskeleton with the membrane is sharply resolved and accurately marks the outer margin of the cytoplasm (**Fig. 7A**, red line). In contrast, the membranes of cells freeze-substituted in acetone/osmium tetroxide (OS) or acetone/glutaraldehyde (GA) are highly contrasted, such that the densely stained outer layer (**Fig. 7A**, light blue ring between red and green circles) enveloping the cytoplasm (gray) is well delineated. The difference between the inner and outer radii of circular cross-sections (**Fig. 7A**, *Δd/*2) is therefore a good estimate of the membrane thickness (*t_memb_*). We measured the diameter of only circular or nearly circular cross-sectional profiles with a sharp cytoskeletal edge present at the cytoplasm/membrane interface. The longest and the shortest orthogonal axes were averaged.

**Fig. 7B** compares histograms of the distribution of outer diameters of circular cross-sections of cells freeze-substituted under the three different regimes. Taking the overall thickness of the membrane, *t_memb_, as Δd*/2, and the average diameter of a *Spiroplasma* cell as *d*∼188 nm (from EM) [14], [19], the membrane thickness would be ∼20 nm. This compares well with estimates from EM tomography of 15–16 nm [18]. The overall thickness of the membrane, regardless of the method of measurement, is unusually high. For comparison, the average thickness of Gram negative bacterial cytoplasmic membranes is only ∼8 nm while that of the complex outer membrane of a typical Gram positive bacteria is ∼11.5 nm.

### 5. Mass and Spatial Organization of Cytoskeleton

Extensive studies on the molecular structure, cellular organization and dynamics of the *Spiroplasma* cytoskeleton, both isolated and in intact cells, point to a complex, multi-component structure (18, 19). The dominant cytoskeletal protein is the 59 kDa Fib protein [21], although other common bacterial proteins such as MreB [18], [20], FtsZ [38], FtsA [39] and elongation factor Tu (EF-TU) [40] are known to be present and may well be part of the cytoskeletal complex. The defining structural component of the cytoskeleton is a helical ribbon, composed mainly of Fib, which is tightly attached to the inner face of the cell membrane and also serves as a linear motor. 2D gels of fractions purified by a variety of complementary procedures reveal a stable cluster of ∼30 peptides that co-purify with the ribbon [18]. This polypeptide cluster is likely to play an important role in the association of the ribbon with the membrane.

The cytoskeletal ribbon of total width ∼70 nm, consisting of seven ∼10 nm-wide parallel fibrils, follows the shortest helical line *L_S_* along the inner surface of the cell membrane [8], [19] (**Fig. 1**, blue line; at the center of the ribbon, *L_R_*). Note that the center of the ribbon need not follow the shortest helical line (unpublished data).

The ribbon length, *l_R_*, per helical repeat *P* (following the shortest line, *l_S_*) is given by:
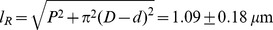
(19)


The ribbon length *L_R_* in an average cell with *n* = 4 turns is
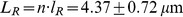
(20)


**Figure 8:**
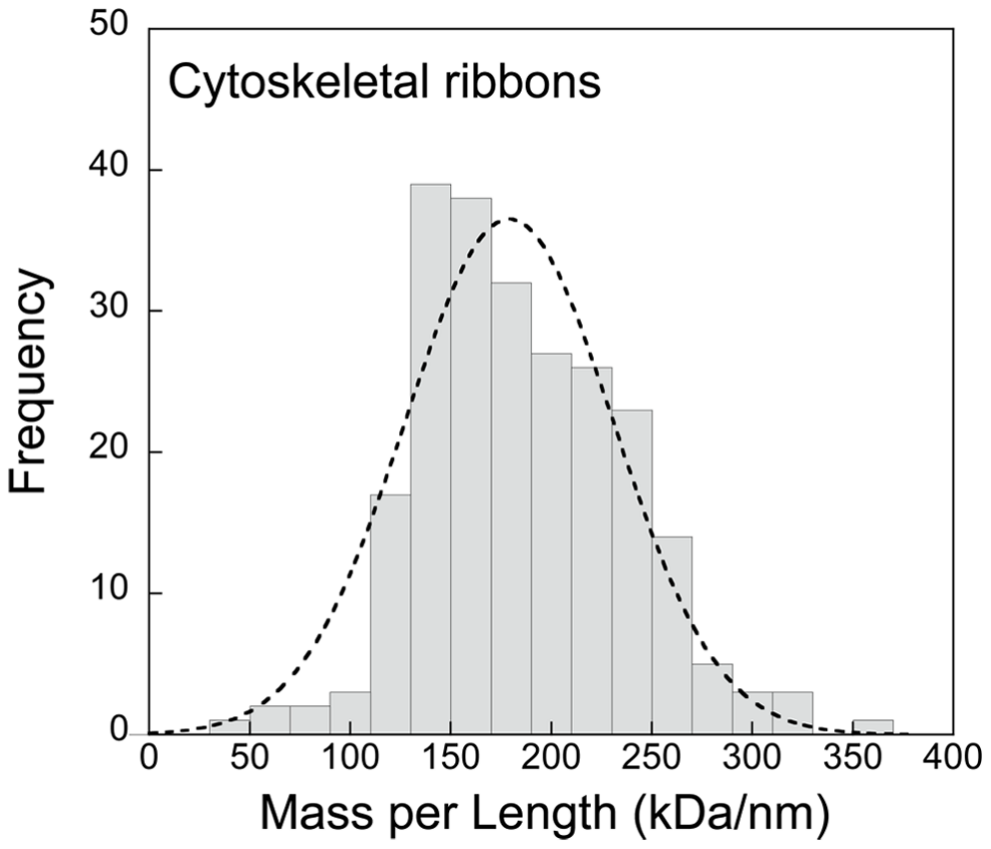
A histogram of the mass-per-length distribution for *Spiroplasma* cytoskeletal ribbons. A Gaussian fit (r^2^ = 0.932) is superimposed on the histogram suggesting a normal distribution. The average linear mass density is 189±70 kDa/nm (SD).

From the average mass-per-length *m_R_* of the cytoskeletal ribbon, which is known from earlier STEM data [16] to have a value of 186 kDa/nm (**Fig. 8**; **Table 1**), we can estimate the total cellular mass of the cytoskeletal ribbon as
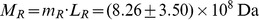
(21)where the error, which is expressed as a standard deviation (as for all the measurements in this study), is mainly due to statistical shot noise in the STEM mass determination of the cytoskeletal ribbon. The uncertainty in the mass-per-length of the isolated ribbons could be estimated from the standard error of the mean, which was around 2% or about ±4 kDa/nm [16].

The cellular mass fraction *f_R_* of the ribbon can now be estimated as:
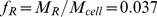
(22)


The number of Fib molecules expected in an average cell can now be estimated in two independent ways. First, using previous data [14], [19] and considering seven fibrils per ribbon assembled as tetramers with an axial repeat of ∼9 nm/tetramer determined by diffraction [16], [19], we can estimate the number of Fib molecules per cell as 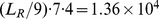. Secondly, the cellular mass of the ribbon divided by the mass of Fib monomers, which is known from sequence data [21], provides an estimate of the number of Fib molecules per cell as 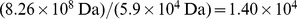 molecules. The consistency between these independent determinations provides confidence about the accuracy of mass of the cytoskeletal ribbon.

### 6. *Spiroplasma* Genome: its Size and Expression

Having analyzed the mass and structural organization of the membrane and its attached cytoskeletal ribbon, we now turn to the remaining compartment, the cytoplasm. The only structural features in the cytoplasm of *Spiroplasma* cells that are clearly visible and identifiable by electron microscopy in thin sections [19] and tomograms [18] are ribosomes and thin filaments thought to represent the strands of DNA that constitute the genome. Extra-chromosomal plasmids also constitute part of the *Spiroplasma* DNA [24]. It is of fundamental interest to know the size and thus the potential complexity of the *Spiroplasma* genome. The molecular mass of DNA in a cell can be estimated from the cell mass 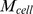 and the dry mass fraction of DNA, which was determined spectroscopically from freeze-dried cell pellets as 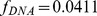. The estimated mass of cellular DNA is therefore:
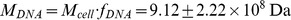
(23)


Since the molecular mass of one DNA base pair (bp) is ∼660 Da, the content of double-stranded DNA chromosome in a single *Spiroplasma* cell is estimated as 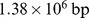. This calculation of DNA mass per cell neglects the impact of ongoing DNA replication, which could lead to overestimation of the average mass of cellular DNA factor by as much as 50%.

This determination compares well with the limited information currently available for *Spiroplasma* genomes [22]–[25]. Of the 23 well-defined groups in the genus *Spiroplasma* [41], 20 genome sizes were estimated by pulsed field gel electrophoresis, and these ranged from 0.98×10^6^ bp (*Spiroplasma* sp w115) to 2.2×10^6^ bp (*S. ixoides* Y32). In particular, the genome of *S. melliferum* BC3, the bacterium that we consider in the present study, was estimated to be 1.46×10^6^ bp.

**Figure 9:**
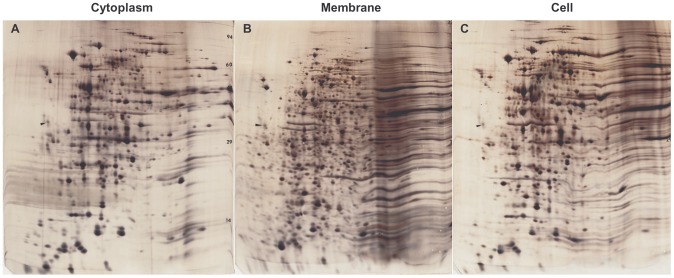
Gene expression. 2D gels of cytoplasm **[A]**, membranes **[B]** and whole cells **[C]**. Gels were aligned and scaled using 20 common spots on gel [**A**] as a reference. About 600 spots can be counted. The cytoplasm comprises about 25% of the mass. Compare to **Table 2** **,** which presents quantitative chemical analysis of unmodified proteins as well as the post-translationally modified glycoproteins and lipoproteins.

Taking the 4,486 genes of *E. coli* as a reference standard, we can estimate that the 1.46×10^6^ base pairs of *S. melliferum* represents ∼1,460 proteins, which provides an estimate of genome reduction in this organism. Genome size sets the upper limit of protein production, although gene expression at any given time is typically much lower. High-resolution, high-sensitivity 2D gels give a reasonable estimate of the extent of gene expression. A conservative count of the number of spots on silver-stained gels extracted from whole *Spiroplasma* cells (**Fig. 9**) is ∼600 (this is in good agreement with 512 expressed proteins found by proteomic analysis of strain KC3 [22]). This suggests that somewhat fewer than half of *Spiroplasma* genes are expressed at a given time under our non-limiting culture conditions. The gels represent the soluble (cytoplasm; **Fig. 9A**) and insoluble (membrane; **Fig. 9B**) compartments as well as whole cells (**Fig. 9C**). Although obvious bleeding of components between fractions occurs, the cell envelope, as indicated by mass analysis (see **Results** **section 4**) contains the bulk (∼0.75) of cell constituents. Therefore, soluble proteins, including all the soluble enzymes needed for physiological functions, account for only a small fraction, ∼0.25, of the cell’s mass.

Further reduction of minimal genomes is possible and does not affect cell viability. In *M. genitalium* with 517 genes of which 480 are protein-coding, about 100 were found to be non-essential [42], [43]. **Table 2** summarizes estimates of cellular components in terms of post-translational and metabolic products of the genome. Given the parasitic lifestyle of *Spiroplasma*s**,** the origin of some of these products is directly from the host or medium.

### 7. *Spiroplasma* Gene Expression: Number of Ribosomes and RNA Content

A separate measure of gene expression and biosynthetic activity can be gleaned from information about the disposition of ribosomes and RNA. The approximately 20-nm diameter of the ribosome makes these nucleic acid/protein assemblies large enough to visualize by TEM. It is therefore possible to determine the number of ribosomes per unit volume 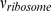 from the number of ribosomes counted in a unit area, 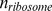, in TEM images of thin sections. We applied a stereological approach [44] with corrections for the Holmes effect [45] as well as for section mass loss [46]. We used rapidly frozen cells that were freeze-substituted in acetone/uranyl acetate, for which ribosomes are highly contrasted in TEM [19].

The number of ribosomes per unit volume is given by
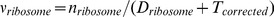
(24)Where 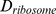 is the ribosomal diameter and 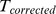 is the section thickness corrected for mass loss and shrinkage. The section thickness determined from clear vertical folds – representing twice the apparent thickness of the adjacent flat section – was estimated to be 45 nm. From our observation of a 50% shrinkage factor perpendicular to the plane of sectioned cells embedded in Spurr’s resin [47] when the sections are irradiated in the electron beam [46], we therefore deduce that the pre-irradiated section thickness *T_corrected_* was 90 nm. Our measurements on TEM images of cryofixed and freeze-substituted cells gave 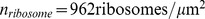 and 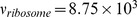 ribosomes/µm^3^. Each ribosome has a volume 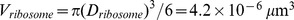, so that the fraction of the cell volume occupied by ribosomes is:



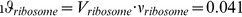
(25)This is in good agreement with tomographic data [48] but lower than the value of 0.08 for *E. coli*. Thus the ribosomes occupy about half as much cytoplasmic volume in *Spiroplasma* as in *E. coli*, suggesting that the biosynthetic machinery, although functionally intact, is not only scaled down but less productive, thus possibly affecting growth and metabolism rates. Note that the ribosome numbers calculated here are for non-limiting culturing conditions; values may be different under limiting growth conditions.

For an average *Spiroplasma* cell volume 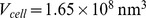 (see **Table 1**), we can estimate the number of ribosomes per cell as
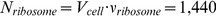
(26)


From the known mass of prokaryotic ribosomes, 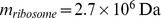, we estimate that the total ribosomal mass per cell is
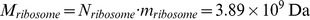
(27)


Most of the cellular RNA is ribosomal, and approximately half of the ribosomal mass is RNA**.** Therefore the RNA content of a cell is 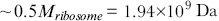, or 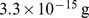. It follows that the mass fraction of RNA in a freeze-dried, intact cell pellet is as 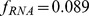.

Taking an average molecular weight of 330 Da for an RNA nucleotide, we estimate that there are 5.9×10^6^ bases of single stranded rRNA per cell.

We can also determine the number of ribosomes per cell directly from chemical analysis of freeze-dried pellets of intact cells. From our determination of total cell mass, 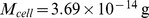, and the mass fraction of RNA in freeze-dried cell pellets, 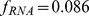; (**Table 2**), we obtain an RNA content of 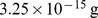 per cell, which corresponds to 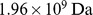 or 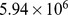 bases per cell. The RNA content obtained from the biochemical analysis is therefore very close to that obtained from the structural determination of rRNA content, suggesting that most of Spiroplasma’s RNA is rRNA. This implies that there are relatively small amounts of mRNA and tRNA.

## Discussion

The mollicutes (Mycoplasmas, Acholeplasmas and Spiroplasmas) represent minimal independent and self-replicating forms of life. On a phylogenetic scale [4], [56] of regressive evolution, the Acholeplasmas and Spiroplasmas are the closest to their clostridial ancestors and have the least reduced genomes. This might explain the relatively large size of Spiroplasmas, their spatial organization and complexity compared to the Mycoplasmas. Spiroplasmas are of particular interest because they include agricultural pathogens of major economic importance such as *S. citri* affecting citrus plants [49] and *S. kunkelii* affecting corn [50], [51] **,** as well as members that have been claimed to be involved in neurodegenerative diseases [52]–[54]. However, we have a different motivation for studying *Spiroplasma*, namely our interest in establishing *Spiroplasma* as a model for analytically studying spatio-temporal, biomechanical and motility aspects of a minimal cell system [55]. To further this goal, we have combined data originating from light microscopy, geometrical measurements, TEM imaging, STEM mass analysis, as well as hydrodynamic and biochemical determinations to establish a quantitative framework for future work.

While spiroplasmas are unique organisms in many ways, and do not represent the bacterial mainstream, their appeal as a model system is greatly facilitated by their inherent helicity. Helicity is a readily determinable geometrical property common in biology. At the cellular level, deviations from helical symmetry, such as those underlying cell motility, can be studied by light microscopy and correlated with local molecular structure as determined by electron microscopy. The simplicity of spiroplasma’s cell envelope, composed of a single unit membrane with a well characterized motor attached to its inner surface, allows straightforward interpretation of the motor’s action, as well as of geometrical and/or motile responses to internal and external cues. Bacterial model systems such as *E. coli, S. typhimurium,* and *C. crescentus* are well characterized genomically and allow the application of sophisticated molecular biology tools. However, they have rather fixed geometries, and rigid and complex envelopes, interiors and motors. Even the helical spirochetes owe their helicity to flagella rotating within the periplasmic space of a complex cell envelope. All things considered, Spiroplasmas represent a blend of physical, structural, molecular, dimensional and dynamic properties that, together with their simplicity and emerging genomics, recommends this organism as a potentially powerful model system.

Interestingly, the free-living, minimal marine bacterium *Pelagiobacter ubique* [57] has a genome size (1.31×10^6^ bps) similar to that of a typical *Spiroplasma*. This may suggest that there is a minimal genome size that allows for full functionality and adaptability in very different environments. Native genomes smaller than *M. genitalium* (the smallest mollicute with a size of 5.77×10^5^ bps) [42] have been reported, e.g., *Nanoarchaeum equitans* (0.49×10^6^ bps) [58] and *Buchneria* sp. APS (0.64×10^6^ bps) [59]. However, unlike the mollicutes, these are obligatory symbionts and are not “free-living”. Mycoplasmas are another candidate for a minimal cell model. They are small and simple enough to address issues related to synthetic genomes [60]–[62], and they are used extensively in systems biology for correlating genome and proteome with cell physiology and metabolism [63]–[65] **.** Although recent work is elucidating the structure of the gliding machinery that is attached to the cell membrane and cell surface [66], mycoplasmas are too pleiomorphic to address fundamental structural questions in a correlative way.

*Spiroplasma*s are optimal for correlating a minimal cell genome and molecular machinery with detectable and quantifiable spatiotemporal events at the whole cell level. The minimal size, content and genome of native mollicutes potentially link them to underlying aspects of synthetic biology. One of the aims of synthetic biology is to create, using a bottom-up approach, an artificial cell by assembling a functional system of subcellular components bound to or enveloped by a membrane system [67]. The bottom-up approach to synthetic cells allows the insertion of anchor, motor and cytoskeletal proteins into artificial vesicles as well as expressing simple complexes [68], [69]. On the other end, the top-down approach envisions reducing an existing native cellular system to a limited set of viable subcomponents [55]. The mollicutes, already minimal forms of life, are ideal vehicles for this approach. The manipulation, reduction and interspecies transfer of whole Mycoplasma genomes is already possible [43], [60], [61]. However, for studying well-defined sub-systems such as cytoskeletons and motor proteins, *Spiroplasma*s might be more suitable. The key to such an approach is a correlated knowledge of the spatial organization of the cell, expressed analytically in geometrical terms [14]–[16], [19], together with its genome products, as we have attempted to demonstrate in this study.

Having described our biophysical measurements to determine the composition and organization of the major cellular components of *Spiroplasma* in terms of a molecular inventory, we can now discuss ways in which the results pose interesting questions about how these cells function as a minimal life form. Although *Spiroplasma* is free living, its parasitic life cycle and consequent genome reduction require some of its biomolecules to be obtained directly from the host organism or growth medium. In addition, the extracellular environment of *Spiroplasma* is partially controlled *in vivo* by the host through a compositionally defined medium, which might reduce the need for specialized machinery to regulate its internal environment. The resulting decreased genome size and smaller number of expressed proteins enables the cellular volume to be greatly reduced since the DNA content is smaller and fewer ribosomes are needed to maintain metabolism. However, along with the reduction in the volume of *Spiroplasma* comes an increase in the ratio of membrane area to cell volume, making it necessary to retain a sufficient number of ribosomes and sufficient cell volume and length to synthesize the protein channels and transporters that are an integral part of the membrane. The minimum surface area of *Spiroplasma* is also determined by the maximum allowed membrane curvature, as well as by the minimum cellular length that is required to accommodate its cytoskeletal motor and force generation**.** The sum of the masses of the major components of *Spiroplasma* is consistent with our measurements of the total cellular mass (membrane and associated proteins, 75±5% (±SD); ribosomes, 19±2%; cytoskeleton, 5±0.5%; DNA, 4±0.6%; since each of these determinations was performed independently, the sum of the measurements is not exactly 100%). Thus, most of the cellular mass is accounted for by the membrane and associated macromolecular complexes.

## Materials and Methods

### 1. Strains, Media and Growth Conditions

*Spiroplasma melliferum* BC3 cells were grown from freeze-dried stock as described in [19] **.**

### 2. Freeze–substitution and Thin Sectioning

Freeze-substitution of whole cells, for ribosome counts and membrane thickness estimations, was carried out as described [70], [71] or in a CS-auto unit (Reichert, Cambridge Instruments). The substitution fluids were acetone containing 2% osmium tetroxide or 2% glutaraldehyde or 2% uranylacetate [18], [19].

### 3. Intact Cell Preparations for STEM Analysis

Cells for STEM mass measurement studies were washed twice in 10 mM Tris, pH 8.0, containing 400 mM NaCl. The cells were processed as described in the **STEM** section below.

### 4. Membrane Vesicle Preparation for STEM Analysis

Cells were pelleted from the medium (Sorval-GSA; 5000 rpm; 4°C; 30 min.) and re-suspended in 10 mM Tris, pH 8.0, containing 400 mM NaCl or 7% sorbitol to maintain iso-osmolarity. The rinsed cells were pelleted (Sorval-SS34; 10,000 rpm; 4°C; 30 min.) and re-suspended twice to remove medium remnants. The final pellet was osmotically lysed by suspension in double distilled water (DDW;37°C) containing DNase and protease inhibitor (Complete Mini;Roche). The cell lysate was sonicated using a thin immersed probe (Kontes micro ultrasonic cell disrupter, 40 W), in short bursts, for 2 min, over a mixture of ice and water. Whole cells and large debris were removed by centrifugation (Sorval SS34; 5,000 rpm; 4°C). The supernatant, containing membrane vesicles, was pelleted (Sorval-SS34; 15,000 rpm; 4°C) and resuspended in DDW. The final supernatant, containing the cytoplasm, was centrifuged at 100,000×g and its supernatant freeze-dried for 2D gel analysis.

### 5. Cytoskeletal Preparations for STEM Analysis

Cytoskeletons were prepared following the procedures described in [15], [18], [19] **.**

### 6. Transmission Electron Microscopy (TEM)

Negative staining, vitrification, freeze-substitution, data collection and analysis were carried out as described in [15], [16], [18], [19] **.** Electron micrographs were recorded using an FEI Tecnai TF30 TEM, operating at an accelerating voltage of 300 kV, and equipped with a Gatan Ultrascan 2 k×2 k pixel CCD camera. An FEI CM200 TEM (with FEG source) and an FEI CM120 TEM (with LaB_6_ source) were used for low-dose cryo-electron microscopy, for which images were recorded on Kodak SO-163 photographic plates.

### 7. Scanning Transmission Electron Microscopy (STEM)

For STEM mass mapping of intact *Spiroplasma*, isolated membranes or cytoskeletons, 5-µl aliquots of suspended cells at a concentration of 1 mg/µl were applied to thin (∼3-nm) carbon films supported on lacy Formvar-carbon films over 200-mesh copper grids. Subsequently, a 2-µl aliquot of tobacco mosaic virus (TMV) at a concentration of 0.4 mg/µl was injected into the drop and also allowed to adsorb for 4 min before being washed 10 times by applying drops of deionized water and drawing off the excess liquid. Grids were partially blotted to maintain a thin layer of water and were immediately plunge-frozen into liquid ethane at −180°C with a KF80 freezing device (Leica). Annular dark-field scanning transmission electron microscope (STEM) images were acquired with a HB501 STEM (VG Microscopes) equipped with a high-brightness field-emission electron source, and were analyzed using NIH Image software as described previously [16]. In brief, images containing 1024×1024 pixels were acquired at an accelerating voltage of 100 kV using a Gatan Digiscan system. The probe diameter was approximately 1 nm, the probe current was adjusted to 2 pA, and a dwell time per pixel of 100 µs was selected, which resulted in an electron dose of approximately 10^3^ electrons/nm^2^, i.e., sufficiently low to avoid significant mass loss due to beam damage. The main source of error in mass determination by STEM is due to counting statistics in the annular dark field images, and slight non-uniformity in the freeze-drying of the cells and cellular components in the vacuum of the electron microscope [29]. In general, the annular dark-field signal is approximately proportional to Z^3/2^, where Z is the atomic number. Errors due to variations in average elemental composition, *i.e.,* variations in average Z, are estimated to be on the order of 5% or less. Such errors are small because the minor elements (P, S, Cl, K and Na) are present at concentrations below 1%, and because the ratios of the major elements (C, O, N and H) do not vary significantly between cellular components, with H contributing a very small fraction of the total annular dark-field signal.

### 8. Statistical Analysis of Helical and Mass Parameters

Spiroplasma cultures are asynchronous, resulting in cells that are variable in size and age. Although it is possible to separate a population of *Spiroplasma* cells into about 12 subpopulations that can be attributed to different growth phases with the median subpopulation most likely associated with the mid-logarithmic growth phase [14], in this study we have analyzed the entire population of cells to avoid bias. The mean values of measured helical parameters for the entire cell population are similar to those for the median subpopulation, but the variances (or standard deviations) are larger. Most geometrical parameters were determined for individual cells and the entire population averaged. For parameters obtained from a product of independent geometrical and mass variables, X and Y, the SD was calculated as:
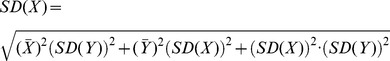



The cell’s volume, mass, and membrane area, were calculated for a standard cell consisting of four helical turns; this corresponds to a median subpopulation that was observed experimentally.

### 9. Stereology

Stereological analysis [44] **of** thin sections of frozen/freeze-substituted cell pellets was used to estimate the number of ribosomes per unit volume. To estimate the volumetric density of ribosomes from the measured number of ribosomes per unit area, it was necessary to make corrections for (1) beam damage, which reduced the pre-irradiated specimen thickness by a factor of approximately two, and (2) the Holmes effect [45], since the pre-irradiated specimen thickness was only about four times the ribosomal diameter. Section thickness was estimated from folds by assuming twice the thickness of the adjacent flat area, and the measured value was corrected for beam damage that is typical for Spurr’s resin [47] due to electron irradiation [46].

### 10. Hydrodynamic Methods: Dynamic Light Scattering (DLS) and Analytical Ultracentrifugation (UAC)

We studied live cells in (i) full medium, (ii) medium without the partially defined organic component (inactivated horse serum, yeast extract, tryptone, peptone and heart-infusion-broth) or (iii) isotonic buffer (phosphate buffer containing 400 mM NaCl). The results between the media did not differ significantly and we report here the results regarding cells in isotonic buffer.

Sedimentation velocity studies were conducted in an Optima XL-I/A analytical ultracentrifuge (Beckman Coulter, Fullerton, CA). Spiroplasmas were suspended in phosphate buffered saline at a final concentration of 1.53 mg/ml as determined by densitometry, using a mechanical oscillator instrument (Anton Paar, Graz, Austria). 400 µl of stock solution, as well as 10 and 100-fold dilutions were loaded into Epon double-sector centerpieces and accelerated to a rotor speed of 3,000 rpm, at temperatures of 9°C and 15°C, respectively. The evolution of sedimentation profiles was recorded using the interference optical imaging system for monitoring refractive index gradients. Dynamic light scattering data were acquired at an angle of 90° at a wavelength of 633 nm, with samples thermostated at 20°C and correlated with a BI-9000 AT autocorrelator (Brookhaven Instruments Corp., Holtsville, NY).

Analysis of the primary data was performed with the software SEDFIT. The sedimentation velocity data were fitted with a distribution of Lamm equation solutions for non-diffusing species, ls-g*(s) [72], combined with systematic noise decomposition [73] **.** In order to determine an average translational diffusion coefficient, the autocorrelation data from the dynamic light scattering data, g^(2)^ (τ), were modeled as a single-exponential decay for a single ideally diffusing species, neglecting potential effects from rotational diffusion or from activities of the cell. A Stokes-radius distribution was calculated with the maximum entropy method [74] as implemented in the software package SEDFIT. Buffer and temperature corrections were performed using the software package SEDNTERP, kindly provided by Dr. John Philo, Alliance Protein Laboratories, using values for the density of 1.00741 g/ml and viscosity of 1.157 Pa s under conditions of the centrifugation experiments, and a viscosity value of 1.0183 Pa s during dynamic light scattering.

The buoyant mass per *Spiroplasma* cell was determined from the measured sedimentation coefficient and known diffusion constant by using the Svedberg equation ([75]; **see Eq. 9**). Cells were assumed not to deform during sedimentation, so that the frictional coefficients for sedimentation and diffusion were considered to be identical.

### 11. Analytical Procedures

#### a. 2D-gel analysis

Whole cells, detergent soluble (membranes) and detergent insoluble (cytoskeleton) fractions were processed by Kendrick Labs (Madison, WI) as described in [18]. The gels were digitized and the images scaled and aligned using a combination of ImageJ [76] and Fiji [77] based on a set of 20 common reference spots.

#### b. Cell component isolation

***Cell preparation*** – Approximately 500 ml of Spiroplasma cell culture was sedimented by centrifugation at 10,000×g for 20 minutes at 4°C followed by five washes in 10 mM Tris, 400 mM sodium chloride buffer, pH 7.5, to remove contaminating growth medium. The supernatant from the last wash was examined for the presence of growth medium by capillary zone electrophoresis (CZE) on a Prince Technologies Crystal 660 Capillary Electrophoresis system equipped with a scanning wavelength array detector (Amsterdam, Netherlands). Separations were performed using a 75-µm i.d., 100-cm long (80-cm to the detection window) fused silica capillary (Polymicro Technologies, Phoenix, AZ) at 220 µA constant current with continuous cooling. The running buffer was 100 mM sodium phosphate buffer, pH 7.5. Once the cell supernatant was shown to be free of growth medium, the organisms were lysed for further analysis.

***Protein precipitation*** – Total protein was precipitated from the cytosolic and solubilized membrane vesicle preparations by using the ProteoPrep protein extraction system (Sigma Aldrich, St Louis, MO). A 1∶1 ratio of 0.2% deoxycholate and sample were mixed together and allowed to stand at room temperature for 10 min. An equal volume of 70% trichloroacetic acid was added and the mixture vortexed for 1 min. The reaction was allowed to incubate at room temperature for 15 min before centrifugation at 15,000×g for 10 min in a microcentrifuge. The precipitate was recovered and redissolved in 100 mM double distilled water.

***Lipid extraction*** – Total lipids were extracted from both membrane vesicles and cytosol by solvent phase extraction. Ten volumes of a 1∶1 v:v chloroform:methanol solution was added to one volume of sample, sonicated in a bath sonicator and incubated at 4°C for 18 hr with constant stirring. The sample was centrifuged at 750×g for 10 min and the supernatant recovered. The extraction was repeated four times, the supernatants pooled and dried in a rotary evaporator.

***Nucleic acid extraction*** – A nucleic acid fraction was prepared by precipitation from the cytosol and solubilized membrane preparations. This was achieved by adding 2.5–3 volumes of 95% ethanol/0.12 M sodium acetate to the sample in a 1.5 ml microcentrifuge tube. The tube was mixed by inversion and incubated in an ice water bath for 10 min. The sample was centrifuged at 12,000 rpm in a Sorvall Fresco microcentrifuge for 15 min at 4°C before removing the supernatant, and drying the precipitate. The dried nucleic acid preparation was reconstituted in double distilled water prior to further manipulation.

#### c. Spectrophotometric analysis of major cell components

***Protein*** – Total protein, irrespective of the subtype, was measured using the fluorescamine assay [78]. In brief, a 1∶1 (v:v) solution of fluorescamine reagent (2 mg fluorescamine dissolved in 10 ml acetone) was added to each sample, which had been buffered to pH 9.0 by the addition of 0.2 M sodium borate, vortexed, and incubated at room temperature for 10 min. The reaction was read in a Turner PicoFluor microfluorimeter (Turner Biosystems, Sunnyvale, CA) at 390_ex_ and 460_em_ and the concentration calculated by comparison against a standard curve.

***Lipid*** – Total lipid content was assayed by mixing a 1∶1 volume ratio of a 1% alcohol solution of Oil Red O dye with the sample and vortexing for 5 min at maximum speed. The sample was spun at 10,000×g for 2 minutes to remove particulate matter and the absorption of the dyed lipid read in an UltraSpec 3300 UV-Vis spectrophotometer (Amersham Biosciences) at 518 nm. The concentration was calculated by comparison to a standard curve.

***Carbohydrate*** – Total carbohydrate content of both cytosol and solubilized membrane fractions was assayed by the technique of Masuko et al [79]. Fifty µl samples were placed into a 96-well microplate to which 150 µl of concentrated sulfuric acid was rapidly added. The mixture was shaken for 30 min before 30 µl of 5% phenol in water was added and the mixture heated for 5 min at 90°C in a dry bath. After cooling to room temperature, reaction product was measured at 490 nm in a BioRad Ultramark EX microplate reader (BioRad Laboratories, Hercules, CA). The concentration of total carbohydrate was calculated by comparison to a calibration curve.

***Nucleic acids*** – Total nucleic acid was directly measured at 260 nm in an UltraSpec 3300 UV-VIS spectrophotometer (Amersham Biosciences, Piscataway, NJ). Before assaying total double stranded DNA (dsDNA), RNA was removed by treatment with RNAse followed by size exclusion chromatography using a Bio-spin Bio-gel P-30 spin column (Bio-Rad, Hercules, CA) to remove degraded RNA. The remaining dsDNA fraction was mixed in a 1∶1 ratio with PicoGreen (Molecular Probes, Eugene, OR) and incubated at room temperature for 5 min before assaying emitted fluorescence from 2 µl of mixture in a NanoDrop ND-3300 fluorospectrophotometer (NanoDrop Technologies, Wilmington, DE). The concentration of dsDNA was calculated by comparison to a standard curve. Total RNA was measured by reacting the nucleic acid isolate with RiboGreen (Molecular Probes), essentially as described for PicoGreen.

#### d. Separation and analysis of component subtypes

***Protein*** – Fractionation into lipoprotein and glycoprotein subtypes was achieved by injecting total protein samples into a MicroTech Scientific X-Treme Simple multi-dimensional micro-HPLC system (MicroTech Scientific, Vista, CA) equipped with Probot Micro-fraction collector (LC Packings). Samples were passed through a 150-µm i.d. C_18_ reverse-phase column. Individual peaks were collected in skirted PCR microplates and subjected to lipid and carbohydrate analysis.

***Lipids*** – The isolation and characterization of phospholipids, glycolipids and cholesterol was achieved by HPLC on normal phase columns. Total lipid extracts were dried in a rotary evaporator, reconstituted in hexane/methyl t-butyl ether (100∶5 v:v) and injected into a 100 µm i.d. x 20-cm normal-phase micro-bore column (Unimicro Technologies, Pleasanton, CA). The different species were isocratically eluted using a hexane/methyl t-butyl ether/acetic acid (100∶5∶0.02 v:v:v) buffer on a LC Packings Ultimate HPLC system, Samples from the HPLC were collected and analyzed for protein and carbohydrate content. Fatty acid species were isolated on a Clarus 500 gas chromatograph (PerkinElmer, Wellesley, MA) using an AT-FAME 30 m×0.25 mm i.d. cross-linked polyethylene glycol fused silica gas chromatography column (Altech Associated, Deerfield, IL). Separation was performed with a temperature program of 150 to 260°C with helium as the carrier gas.

***Carbohydrate*** – Free carbohydrates were isolated by lectin affinity chromatography using a 25 lectin affinity array. Each lectin phase was produced by immobilizing commercially available biotinylated lectins (Sigma Aldrich) onto streptavidin-coated magnetic particles (BioRad) placed into 25 different wells of a microtiter plate. A sample was incubated with each of the different lectin-coated beads, separated by a magnetic separator (BioRad), washed five times in 100 mM sodium phosphate buffer, pH 7.4, and the bound carbohydrate released by incubating the beads with a solution containing an excess of a specific lectin-competing sugar. The beads were again sedimented by a magnetic separator, the resulting supernatant recovered, and competing sugars removed by passing through a Bio-spin Bio-gel P-30 spin column (Bio-Rad). Carbohydrate content of the eluent was measured in a refractometer.
